# High prevalence of syphilis among recyclable waste collectors in Central Brazil

**DOI:** 10.1590/0037-8682-0283-2023

**Published:** 2024-02-12

**Authors:** Wesley Marcio Cardoso, Ana Rita Coimbra Motta-Castro, Sabrina Moreira dos Santos Weis-Torres, Larissa Melo Bandeira, Minoru German Higa, Marco Antonio Moreira Puga, Ana Rita Barbieri, Sonia Maria Fernandes Fitts

**Affiliations:** 1 Universidade Federal de Mato Grosso do Sul, Campo Grande, MS, Brasil.; 2 Fiocruz Mato Grosso do Sul, Ministério da Saúde, Campo Grande, MS, Brasil.

**Keywords:** Epidemiology, Sexually transmitted infection, Syphilis

## Abstract

**Background:**

Syphilis is associated with social and behavioral factors.

**Methods:**

This cross-sectional study determined the prevalence of syphilis and its associated risk factors among recyclable waste collectors in Central Brazil.

**Results:**

A lifetime syphilis prevalence rate of 7.91% (95% confidence interval: 5.25-11.75) was found among 278 participants. Low educational level, history of sexually transmitted infection, and age ≥ 36 years were associated with a high prevalence of lifetime syphilis.

**Conclusions:**

These findings emphasize the need for syphilis prevention, screening, and treatment among recyclable waste collectors, highlighting the potential for the spread of infection in vulnerable populations.

Syphilis is caused by the spirochete *Treponema pallidum* subspecies *pallidum*, which affects the sexual and reproductive lives and well-being of affected individuals, and remains a public health problem^1^. Despite the availability of well-defined diagnostic methods and effective treatments, controlling syphilis is still challenging^2^. 

Interactions between social and behavioral factors can influence the prevalence of syphilis^2^. Scientific literature has reported that specific populations are highly vulnerable to acquiring syphilis, such as men who have sex with men (MSM), sex workers, prisoners, sugarcane cutters, the homeless, and recyclable waste collectors^3^ ^-^ ^8^. Vulnerable populations such as recyclable waste collectors have been shown to contribute to the acquisition of sexually transmitted infections (STI) because of their health, social, behavioral, and environmental deficiencies. Most public health prevention policies associated with these diseases are not accessible to these vulnerable populations^3^.

In Brazil, few studies have investigated the association between risk factors and behavioral patterns of STI susceptibility in this population^3^ ^,^ ^9^ ^,^ ^10^. Considering the sociodemographic characteristics of recyclable waste collectors and the scarcity of studies on syphilis, this study aimed to estimate the prevalence of syphilis and the factors associated with this infection in recyclable waste collectors in Campo Grande, Central Brazil.

This cross-sectional study was conducted between April 2014 and July 2016 in Campo Grande, Mato Grosso do Sul, Central Brazil. The participants included in this study were recruited from six recycling cooperatives and dumping grounds. The inclusion criteria for the study population were male and female individuals aged over 18 years who worked in dumping grounds or organized recycling cooperatives. The required sample size was estimated to be 231 participants based on the prevalence of syphilis (18.4%)^3^ among recyclable waste collectors, considering a 95% confidence interval (CI), 5% margin error, and 20% non-respondents. Of the 727 recyclable waste collectors, approximately 38,3% agreed to participate in the study and had worked at cooperatives and dumping grounds. Participation was voluntary, and participants were informed of the confidentiality of the data and research objectives. After reading and signing the informed consent form, each participant was interviewed face-to-face using a questionnaire to obtain information about sociodemographic characteristics and risk factors associated with syphilis (Supplementary material 1). This study followed the recommendations of the Ethical Committee on Human Research. Therefore, the study protocol was approved by the Ethics Committee of the Federal University of Mato Grosso do Sul (CAAE 23009613.6.0000.0021/13).

Blood samples (10 mL) were collected via a peripheral venous puncture. Serum samples were subjected to a specific treponemal test to determine the lifetime syphilis infection. A commercial enzyme-linked immunosorbent assay kit was used (Syphilis, Diasorin, Italy). The Venereal Disease Research Laboratory (VDRL) test (Wiener lab®, Argentina)*was performed (serial dilution of positive samples)* to determine whether the disease was active. After the test results were obtained, a medical team assisted the participants with active syphilis infection with a positive VDRL test result. Patients were then referred to the primary healthcare system to receive benzathine penicillin G (BPG). All the participants received information on STI prevention.

The collected data were entered into an Online database*Research Electronic Data Capture*. The prevalence rates of lifetime syphilis infections and the 95% CI were calculated. Statistical analyses were performed using*Stata*version 13 (StataCorp LP, College Station, TX, USA). Univariate analysis was performed to verify the association between each independent and dependent variables (positive for anti-*T. pallidum*). The multiple logistic regression model included variables with p-value ≤ 0.20. Statistical significance was set at*p <*0.05.

A total of 350 recyclable waste collectors were invited to participate in the study, of which 278 (79.42%) consented to participate (Figure 1). The ages of the participants ranged from 18 to 70 years (median age: 35.93 years). Fifty percent of the respondents were women (139/278) and 59.71% worked in a dumping ground in Campo Grande City. The age group with the highest number of members was adults aged up to 35 years (58.27%, 162/278). Regarding race/ethnicity, most participants were multiracial, as self-reported by the majority (63.66%, 177/278). Most participants reported regular sexual partnerships (72.7%) and a monthly income of USD 376 or less (56.8%). Of the total participants, 40.3% (112/278) had worked for ten years as a recyclable waste collector.

**FIGURE 1:**
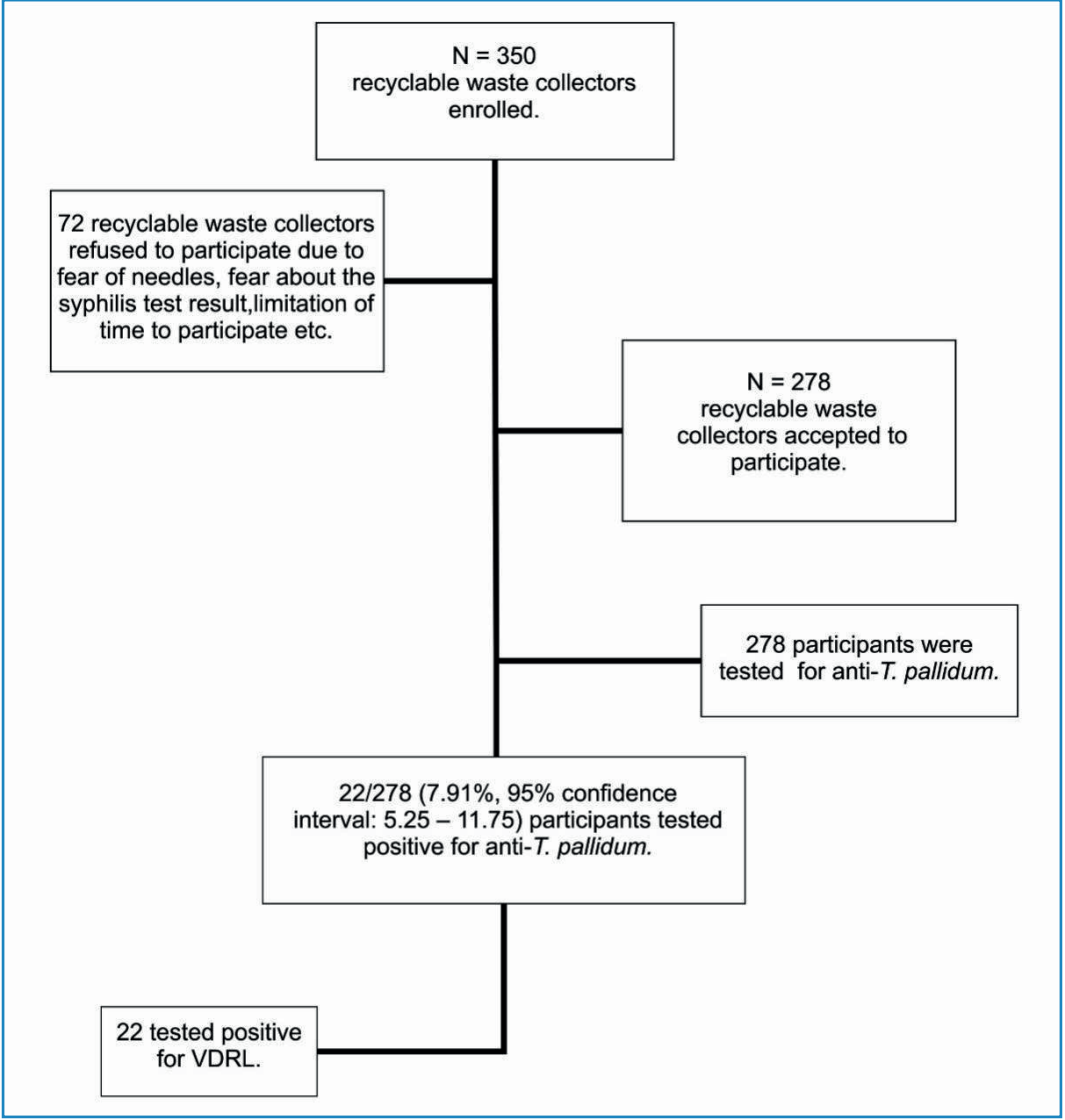
Flowchart of enrollment of participants tested for syphilis in this study. **VDRL:** Venereal Disease Research Laboratory.

Among the interviewed participants, 22 tested positive for the anti-*T. pallidum*(7.91%; 95% CI: 5.25-11.75). Thus, high rates confirmed the prevalence of syphilis, and BPG is the first recommended treatment for syphilis in Brazil^11^.

In the univariate analysis, risk factors associated with the prevalence of syphilis were found in individuals aged ≥ 36 years (independent of sex) (*p ≤*0.001). In addition to age, the incidence of syphilis was higher among recyclable waste collectors who reported a family income lower than $376 than that of their counterparts (*p =*0.004). The prevalence of syphilis was significantly higher in individuals with lower educational levels (*p <*0.001) and those who were illiterate (*p=*0.001) than that of their counterparts. A previous report showed that STIs were associated with a higher prevalence of syphilis (*p*< 0.001). In this study, symptoms such as wounds/genital ulcers in the last year or lifetime were significantly associated with the prevalence of syphilis (*p =*0.007 and 0.002, respectively).

Multivariate analysis revealed that schooling from 1 to 4 years (adjusted odds ratio [AOR] = 4.09 [1.10-15.17], *p* = 0.035), age ≥ 36 years (AOR = 5.7196 [1.507-21.706]), and previous report of STI (AOR = 10.89 [3.37-35.16], *p* ˂ 0.001) were factors associated with a high prevalence of syphilis (Table 1).

**TABLE 1:** Factors associated with lifetime syphilis among recyclable waste collectors in Central Brazil.

Variables	Prevalence of syphilis (%)		OR (95% CI)	Adjusted OR (95% CI)
**Age (years)**				
≤ 35	3/162	1.85	1	1
36-45	9/49	18.37	11.92 (3.08-46.08)	7.68 (1.56-37.65)
46-70	10/67	14.93	9.30 (2.47-34.98)	4.95 (1.06-23.13)
**Schooling (years)**				
5-12	6/197	3.05	1	1
1-4	11/57	19.30	7.65 (2.67-21.65)	4.09 (1.10-15.17)
Illiterate	5/24	20.83	8.88 (2.34-30.04)	3.39 (0.73-15.54)
**Monthly income**				
> 376 USD	11/213	5.16	1	-
≤ 376 USD	11/65	16.92	3.74 (1.54-9.09)	-
**History of STI** ^a^				
No	11/246	4.47	1	1
Yes	11/31	35.48	11.75 (4.53-30.45)	10.89 (3.37-35.16)
**Wound/genital reported during lifetime** ^a^				
No	14/239	5.86	1	-
Yes	8/36	22.22	4.59 (1.77-11.91)	-
**Wound/genital reported in the last 12 months** ^a^				
No	17/256	6.64	1	-
Yes	5/20	25.00	4.68 (1.52-14.44)	-

**CI:** confidence interval; **OR:** odds ratio; **STI:** sexually transmitted infection. ^a^The total represents the number of individuals who answered the question.

This original study is relevant for public health, as it investigates the prevalence and factors associated with syphilis in recyclable waste collectors working in cooperatives and dump grounds in Campo Grande, Central Brazil. In this study, the recyclable waste collector population was considered a poor population, comprising young adults, self-reported multiracial individuals, individuals with regular sexual partners, individuals with a predominance of schooling under 12 years of age, and individuals with a family income of $376 or less. Most individuals have worked as recyclable waste collectors for more than ten years. 

The unfavorable sociodemographic and economic aspects of this population directly influence health conditions. These factors are directly associated with the stigmatizing situation of social exclusion, which causes sanitary, social, behavioral, and environmental deficiencies that ensure high vulnerability to infectious diseases^9^.

In addition, a low educational level is associated with a high prevalence of STIs. This is probably because the level of knowledge interferes with decision-making and the degree of awareness of the danger that an STI can represent. These factors determine the adoption of risk behaviors, such as inconsistent condom use, which favors the spread of these diseases^12^. 

We found a high percentage of lifetime syphilis cases (7.91%; CI 95%: 5.25-11.75). The increase in the number of syphilis cases is associated with health problems worldwide. A substantial increase in syphilis prevalence among MSM revealed that the global syphilis prevalence was 7.5% (95% CI: 7.0-8.0) from 2000-2020^13^. Recyclable waste collectors have a high rate of syphilis. Brazilian Health Regulatory Agency published data similar to studies that reported that the prevalence rates of lifetime syphilis in blood donors were 1.08% and 0.95% in Brazil and Central Brazil, respectively^14^. A higher percentage of lifetime syphilis was found among recyclable waste collectors in Santos City, São Paulo (18.4%; 95% CI: 13.63-23.17)^3^. Considering studies on syphilis conducted in different vulnerable populations, the high prevalence of lifetime syphilis found in this study is consistent with that of a study conducted on prisoners of Mato Grosso do Sul, Central Brazil (10.5%; 95% CI: 9.56-11.67)^15^. These findings demonstrate that interventions targeting high-burden groups, including recyclable waste collectors, are required to control syphilis.

One factor that intensifies the vulnerability of the studied population is that many recyclable waste collectors reside in invaded areas. Thus, they are not part of the coverage of the primary healthcare system in the region. It is worth noting that access to health services contributes to the screening, treatment, and interruption of syphilis transmission. This study provided recyclable waste collectors access to treatment, information on prevention, and healthcare during data collection. Recyclable waste collectors received guidance and multi-professional care from the healthcare team at the Federal University of Mato Grosso do Sul.

After performing multivariate analysis, a higher prevalence of lifetime syphilis was observed in individuals with lower educational levels, a history of STI, and those aged 36 years or older. The lack of knowledge about the signs and symptoms of STIs and difficulty in accessing health services may contribute to syphilis transmission in vulnerable populations. In addition, patients with syphilis and other STIs are often asymptomatic, facilitating the efficient transmission of these infections^12^.

Age more than 36 years was a statistically significant factor (p ≤ 0.001). This direct association may suggest that with increasing age, the risk of acquiring STIs such as syphilis by exposure increases over time^7^. An association found between syphilis and age ≥ 36 years should be interpreted with caution since the majority of the studied population was over 35 years old. Additionally, no cases of syphilis were found in individuals aged 18-27 years.

Another hypothesis that may reinforce the risk associated with exposure time is the cultural characteristics of the history of STI, which are probably due to inconsistent condom use. Although condom use was not associated with multivariate analysis, recyclable waste collectors reported irregular condom use, which increased the risk of exposure and transmission. 

This finding has also provided essential data for health authorities, showing that prevention policies for STIs are not working adequately for young adults.

The present study has some limitations. First, information was obtained through interviews, which allowed the omission of responses mainly related to sexual behavior, resulting in an underestimation of the variables. Second, some information required desirability of the memory of the trial participant, which could lead to difficulty in remembering. Third, as a cross-sectional study, exposure and outcomes were simultaneously assessed. Therefore, it is impossible to infer causality because a temporal sequence cannot be established. Despite these limitations, the experiences provided during the interviews, blood collection, delivery of results, and guidance on healthcare represented a significant opportunity, self-knowledge, and personal growth for the study population. In addition, the results of this study provide relevant data on the prevalence of syphilis in a vulnerable population, presenting unique information that can guide the planning of syphilis control and prevention strategies.
