# Supplementary material for: High prevalence of syphilis among recyclable waste collectors in Central Brazil
Source: Rev Soc Bras Med Trop. 2024 Feb 12;57:e00702-2024. doi: 10.1590/0037-8682-0283-2023 (PMC10911280; doi:10.1590/0037-8682-0283-2023)
Supplement: Supplementary file 1 [file 1678-9849-rsbmt-57-e00702-2024-supp1.pdf]

# STUDY OF LIVING AND HEALTH CONDITIONS OF RECYCLABLE WASTE COLLECTORS IN CAMPO GRANDE, CENTRAL BRAZIL.

## 1. GENERAL INFORMATION

Cooperative ________________________

Recyclable waste collectors at the dumping ground (  ) 1.1 initials: __________

1.2 Name:_____________________________________________________

1.3 Date of birth:___/___/___

1.4 Place of birth:____________________________

1.5 Adress: _________________________House number: ________

Neighborhood:_________________ Phone number:_______________________

1.7 How long have you been living in Campo Grande city______

## 2. SOCIODEMOGRAPHIC DATA

2.1 What is your (self-declared) race/color?

(  ) White (  ) Black (  ) Multiracial (  ) East Asian (  ) Indigenous

2.2 What is your religious affiliation or belief? (  ) Catholic (  ) Evangelical (  ) Spiritist (  ) None (  ) Another religion ______

2.3 Marital Status? (  ) Single (  ) Married/ with a partner (  ) Divorced/ without a partner (  ) Widow/Widower I__I

2.4 Who lives with you?

| **Name / Family Relationship** | **Age** |
|---|---|
| | |
| | |
| | |
| | |
| | |

2.5 How many years did you study? (years of study) ________________________

2.6 Monthly Family Income...............US$____________________

2.6.2 How many people are responsible for this income? I__I__I

2.7 Do you own a house? I__I No I__I Yes

2.7.1 Type of residence: I__I Masonry construction I__I Wooden construction

I__I Canvas shack-type construction

2.7.2 How many rooms are there in your residence? I__I__I How many bathrooms?I__I

2.7.3 Does your house have access?

Sewer system? ( ) No ( ) Yes

Electrical power grid? ( ) No ( ) Yes

Water supply network? ( ) No ( ) Yes

Where does your water supply come from? (origin of drinking water) _________

## 3 WORK ACTIVITY

3.1 Previous job before working as a recyclable waste collector?_________________

3.2 How long have you been working as a recyclable waste collector?_____

3.3 How long have you been working at the cooperative?_______

3.4 Do you engage in any other activity? ( ) No ( ) Yes What?________________

3.4.1 Have you experienced any type of work-related accident as a recyclable waste collector?

( ) No ( ) Yes – What kind of accident?__________________________

3.5 Do you use personal protective equipment? ( ) No ( ) Yes

How often do you use personal protective equipment?

| **PPE** | **Always** | **Sometimes** | **Never** |
|---|---|---|---|
| Gloves | | | |
| Boots | | | |
| Apron | | | |
| Cap | | | |
| Glasses | | | |

| | | | |
|---|---|---|---|
| long-sleeved shirt | | | |
| Others | | | |

3.6 In your opinion, can handling recyclable material cause illness??

( ) No ( ) Yes If yes, which ones?______________________________________

**SURVEY FORM ABOUT RISK FACTORS FOR INFECTIOUS DISEASE**

**1.** Do you have any disease? ( ) No ( ) Yes

If yes, which ones? __________________________________________

**2.** How old were you when you had your first sexual intercourse? _____

**3.** Is there any case of Hepatitis, AIDS, or Syphilis in your family? ( ) No ( ) Yes

If yes, which ones? ___________

**4.** If yes, what is the degree of relationship? ( ) Father ( ) Mother ( ) Husband/wife ( ) Brother/sister ( ) Other____________________

**5.** Have you ever received a blood transfusion? ( ) No ( ) Yes, 1994 or after ( ) Yes, before 1994

**6.** Have you ever had a sexual relationship with someone who has received a blood transfusion? ( ) No ( ) Yes

**7.** Have you ever had any surgery? ( ) No ( ) Yes If yes, what surgery?________

**8.** Have you ever had a tattoo? ( ) No ( ) Yes

**9.** Have you had a piercing? ( ) No ( ) Yes

**10.** Have you shared personal cutting objects such as nail clippers, and razor blades with anyone? ( ) No ( ) Yes

**11.** Have you ever had acupuncture with needles? ( ) No ( ) Yes

**12.** Have you ever had dental treatment? ( ) No ( ) Yes

**13.** Are you currently sexually active? ( ) No ( ) Yes Number of partners ___/last 5 years

**14.** Have you ever had a sexual relationship with a same-sex partner? ( ) No ( ) Yes

**15.** About drug use:

15.1 Have you ever used drugs in your lifetime? ( ) No ( ) Yes

If yes, which ones?_____________

15.2 Have you used drugs intravenously? ( ) No ( ) Yes

15.3 Has a sexual partner or someone with whom you've had sexual relations ever used drugs?

( ) No ( ) Yes If yes, which ones?_____________

**16.** Do you use condoms? ( ) No ( ) Yes

If yes, how often? ( ) Always ( ) Sometimes ( ) Never

**17**. Do you know which diseases condoms help prevent? ( ) No ( ) Yes

If yes, which ones?___________________________

**18.** Do you know what condoms are used for? ( ) No ( ) Yes

If yes, for what? _________________________

**19.** Have you ever had any Sexually Transmitted Infection (STI)?

( ) No ( ) Yes ( ) no/inf.

If yes, when?___________ Which STI?_____________________________

**20.** Have you ever received money for sex? ( ) No ( ) Yes

**21.** Have you ever been incarcerated? ( ) No ( ) Yes

If yes, how many times?_______________

**22.** Have you ever had hemodialysis? ( ) No ( ) Yes

**23.** Do you smoke? ( ) No ( ) Yes If yes, How many cigarettes by day?_________

**24.** About the consumption of alcoholic beverages:

( ) Does not consume

( ) Rarely consumes (< 1 day/week)

( ) Occasionally consumes (1-3 days/week)

( ) Frequently consumes (4 -6 days/week)

( ) Consumes daily

In what quantity? _________________week

**25.** Have you been tested for:

Hepatitis B: ( ) No ( ) Yes

Hepatitis C: ( ) No ( ) Yes

HIV: ( ) No ( ) Yes

Syphilis: ( ) No ( ) Yes

If yes, what was the result?

Hepatitis B: ( ) Negative ( ) Positive

Hepatitis C: ( ) Negative ( ) Positive

HIV: ( ) Negative ( ) Positive

Syphilis: ( ) Negative ( ) Positive

**26.** If you are living with Hepatitis B, C, HIV, or Syphilis have you started treatment?

( ) No ( ) Yes How long?:_______________

**27.** Have you had any genital discharge in the last 12 months?

( ) No ( ) Yes ( ) Do not remember

**28.** Have you had any wounds/ulcers on the genital area in the last 12 months?

( ) No ( ) Yes ( ) Do not remember

**28.1** Have you ever had?

( ) genital discharge ( ) ulcers genital ( ) genital warts ( ) genital blisters

**29.** Do you know the ways of transmission of the Hepatitis B virus? ( ) No ( ) Yes

If yes, specify____________________________________

**30.** Do you know the ways of transmission of Hepatitis C? ( ) No ( ) Yes

If yes, specify: ________________________________

**31.** Do you know the ways of transmission of Syphilis? ( ) No ( ) Yes

If yes, specify: ________________________________

**32.** Do you know the ways of transmission of HIV? ( ) No ( ) Yes

If yes, specify: ________________________________

**33.** Do you know the prevention methods for Hepatitis B virus? ( ) No ( ) Yes

If yes, specify: ________________________________________

**34.** Do you know the prevention methods for Hepatitis C virus? ( ) No ( ) Yes

If yes, specify: ________________________________________

**35.** Do you know the prevention methods for Syphilis? ( ) No ( ) Yes

If yes, specify: ________________________________________

**36.** Do you know the prevention methods for HIV? ( ) No ( ) Yes

If yes, specify: ________________________________________

**37.** Have you been vaccinated against Hepatitis B? ( ) No ( ) Yes ( ) Do not remember

If yes, how many doses?_______________

**38.** Have you been vaccinated against Diphtheria/Tetanus (DTa)?

( ) No ( ) Yes ( ) Do not remember

If yes, how many doses?_______________

**39.** Would you like to receive these vaccines?

( ) No ( ) Yes

If not, why? ________________________________________________

**40.** As a recyclable waste collector, have you had contact with feces?

( ) No ( ) Yes If yes, Which type? ( ) Human ( ) Animal

**41.** Do you ingest food found in recycling? ( ) No ( ) Yes
